# Effectiveness, quality of life, and safety of secukinumab versus conventional systemic therapy in patients with erythrodermic psoriasis: a comparative study

**DOI:** 10.3389/fmed.2024.1473356

**Published:** 2024-11-14

**Authors:** Ying Zhou, Weiquan Chen, Linglu Fang, Fang Qiu, Jiayuan Wu, Jing Li

**Affiliations:** ^1^Institute of Dermatology and Venereal Diseases, Affiliated Hospital of Guangdong Medical University, Zhanjiang, Guangdong, China; ^2^Department of Dermatology and Cosmetology, Dongguan Maternity and Child Healthcare Hospital, Dongguan, Guangdong, China; ^3^Institute of Dermatology, Chinese Academy of Medical Sciences & Peking Union Medical College, Nanjing, Jiangsu, China

**Keywords:** erythrodermic psoriasis, secukinumab, conventional systemic therapy, propensity score matching, real-world evidence

## Abstract

**Background:**

Erythrodermic psoriasis (EP) is a rare but life-threatening variant of psoriasis less responsive to conventional systemic therapies (CST). Limited research exists on the management of EP with secukinumab.

**Objectives:**

To compare the effectiveness, quality-of-life effects and safety of secukinumab versus CST in patients with EP.

**Methods:**

EP patients treated with either secukinumab or CST between August 2020 and October 2022 were identified using the National Clinical Research Center for Dermatologic and Immunologic Diseases (NCRC-DID) database encompassing 962 healthcare organizations. Propensity score matching (PSM) was performed to balance the cohorts based on demographic and clinical characteristics. The primary outcomes assessed were Psoriasis Area and Severity Index (PASI), Body Surface Area (BSA), and Dermatology Life Quality Index (DLQI) scales at 4 weeks, 10–14 weeks, and 22–24 weeks.

**Results:**

The study included 311 patients (160 receiving secukinumab and 151 receiving CST), among them, 101 matched pairs were generated by propensity score matching (PSM). Secukinumab recipients displayed a notably accelerated response compared to those receiving CST, evidenced by significantly higher rates of achieving PASI50 (before PSM: 73.8% vs. 61.6%, after PSM: 76.2% vs. 63.4%), PASI90 (before PSM: 36.9% vs. 25.8%, after PSM: 40.6% vs. 25.7%), and BSA50 (before PSM: 64.4% vs. 50.3%, after PSM: 68.3% vs. 51.5%) at week 4 (*p* < 0.05). However, before PSM, secukinumab showed significantly higher DLQI0/1 rates at weeks 4 (41.3% vs. 29.8%) and 12 (63.8% vs. 44.8%). After PSM, statistically significant differences were observed at week 12 for PASI and BSA scores, and at week 4 for DLQI scores (*p* < 0.05). Similar efficacy trends were observed in other outcomes at week 0 up to week 24, but no statistical differences were noted.

**Conclusion:**

Compared to the CST, secukinumab tend to offer a more rapid response and achieve greater improvements in clinical symptoms and quality of life for EP patients.

## Introduction

Erythrodermic psoriasis (EP) is a rare and life-threatening subtype of psoriasis, representing approximately 1 to 2.25% of psoriasis cases, with a mortality rate reaching 9% (1, 2). Clinically, EP is a condition triggered by certain stimulations, characterized by widespread erythema, scaling, and infiltration that rapidly affects more than 75% of the body surface area (BSA) (3). Urgent management is required to prevent complications, including electrolyte imbalances, malabsorption, anemia, cardiac failure, and sepsis (4, 5). Due to its rarity and complexity, treating EP is challenging and the conventional systemic therapy (hereinafter referred to as CST) was commonly employed including retinoid, cyclosporine, methotrexate. Acitretin and methotrexate suit less acute cases due to slower onset (6, 7). By contrast, cyclosporine monotherapy elicits faster effects compared to acitretin and methotrexate, yet high doses may lead to hypertension and nephrotoxicity (1, 8). Furthermore, CST is often accompanied by treatment failure and the potential risks of blanket immunosuppression, intolerance, visceral toxicity, and opportunistic infections (9). Alternative and innovative options are urgently needed for this fatal psoriatic entity.

Biological therapy (BT) represents an emerging and highly effective therapy in EP, including TNF (tumor necrosis factor)-*α* inhibitors, interleukin (IL)-17 inhibitors, and IL-12/23 inhibitors. A recent systematic review of EP patients treated with various biologics hitherto reported that, out of 122 patients who underwent BTs and provided Psoriasis Area and Severity Index (PASI) scores, 91.0% reached PASI50, indicating the high effectiveness of BT for EP (3). The latest National Psoriasis Foundation Consensus Guidelines recommend infliximab as the only first-line biologic due to limited experience with other biologic drugs at the time (10).

IL-17A is considered a major ‘driver’ of pro-inflammatory cytokines in the pathogenesis of psoriasis, as it can activate keratinocytes and promote their hyper-proliferation (11–13). As the first biologic targeting IL-17 approved for the management of psoriasis, secukinumab has been proven to provide safe, rapid, and sustained improvements in patients with plaque psoriasis, psoriatic arthritis (PsA), and ankylosing spondylitis. Successful management of EP with secukinumab has been described in several case series and reports (10, 14, 15). It may play a positive role in the therapy of EP. As the rarity and severity of EP, which complicates the execution of clinical randomized controlled trials (RCTs), there is insufficient high-level evidence to conclude that secukinumab is a superior substitute for CST in acute and unstable EP patients. Post-marketing registries facilitate the collection of large samples of real-world data from diverse patient populations. As a supplement to clinical trials, comparative cohort studies based on these data can provide evidence to evaluate the efficacy and safety of drugs and offer insights into drug performance in real-life settings (16). The current study utilized the national database of the Chinese Psoriasis Group network and employed the propensity score-based matching method, and aimed to compare the effectiveness of secukinumab versus CST in patients with EP. The study protocol was reviewed and approved by the Ethics Committee of the Affiliated Hospital of Guangdong Medical University (No. KT2023-011-01).

## Methods

### Study design and materials

This is an exploratory analysis of treatment outcomes with secukinumab versus CST. The sample was obtained from the Chinese Psoriasis Group network database of the National Clinical Research Center for Dermatologic and Immunologic Diseases (NCRC-DID)^1^ between the establishment (August 2020) of the database to October 2022. NCRC-DID is a national health research network that provides access to psoriasis registration data from 962 healthcare organizations in China.

### Inclusion and exclusion criteria

Eligible patients satisfied the following inclusion criteria simultaneously: (i) a diagnosis of EP, characterized by diffuse erythema and desquamation affecting more than 75% of BSA. While the traditional definition of erythroderma involves more than 90% of BSA and is widely cited in dermatology, a 75% BSA threshold is sometimes used in EP to identify severe cases requiring prompt intervention (1, 17), thereby ensuring timely and appropriate systemic treatment for patients with significant disease; (ii) having a documented history of diagnosed psoriasis, displaying prototypical psoriatic lesion characteristics clinically, or undergoing histopathological examination confirming psoriasis while excluding other primary differential diagnoses for erythroderma; (iii) undergoing assessments utilizing the PASI, BSA, and Dermatology Life Quality Index (DLQI) scale after receiving treatment at 4 weeks, 10–14 weeks, and 22–24 weeks. Exclusion criteria included incomplete key data and receiving small-molecule inhibitors, PDE4 inhibitors, and combination treatments with both CST and secukinumab. To maximize external validity, comorbid conditions and concomitant topical therapies were not excluded.

### Primary outcomes

According to treatment methods, the patients were divided into secukinumab or CST cohorts (receiving systemic immunosuppressants, retinoid derivatives, and any other conventional medications). The following information of included patients was recorded for analysis: (i) demographic and clinical characteristics, including gender, body mass index (BMI), smoking situation (yes/no/quit), duration since psoriasis diagnosis, family history of psoriasis, and age at treatment initiation; (ii) comorbidities associated with psoriasis, such as cardiovascular disease, diabetes, and rheumatism; (iii) association with PsA, diagnosed by a rheumatologist; (iv) baseline and follow-up (W4, W12, W24) BSA and PASI scores, and the number of patients achieving BSA50/75 and PASI50/75/90, which are defined as a reduction of at least 50%/75% in body surface area involvement and an improvement of at least 50%/75%/90% in the PASI score from baseline, respectively; (v) DLQI score and the count of cases with DLQI0/1 (obtained a DLQI score of ≤1, no effect of psoriasis on QoL) at different time points; (vi) Observed adverse events (AEs).

### Statistical analysis

Continuous variables for baseline characteristics were compared using the paired t test or the Mann–Whitney U test, and categorical variables were analyzed using chi-square test or Fisher exact test. Propensity score matching (PSM) was performed to balance the cohorts. The variables gender, age, BMI, marital status, education level, smoking history, family history, psoriasis duration, previous systemic treatment, PsA, comorbidity and baseline PASI, BSA, and DLQI score were included in the logistic regression model for calculating the propensity scores, with treatment type as the dependent variable. Patients treated with secukinumab were matched 1:1 with patients treated with CST using nearest neighbor matching with a caliper of 0.1 without replacement. All analysis were performed using SPSS 26.0 software (SPSS Inc., Chicago, IL, USA).

## Results

### Study population

A total of 311 patients (160 received secukinumab and 151 received CST) were identified with EP who were recorded with complete data. The pre-PSM baseline characteristics of the two cohorts revealed statistically significant differences in marital status, education level, family history of psoriasis, and PsA (*p* < 0.05), while gender was not as significant but was still important (*p* = 0.061) (Table 1). Post PSM, no statistical differences were observed in any features in each pairwise comparison, confirming the comparability in two cohorts. An additional 109 patients were excluded due to non-overlapping propensity scores, leaving a total of 202 patients for the final analysis. The rest of the baseline characteristics of the population are summarized in Table 1. Because of some cases lost to follow-up at W12 and W24, a complete case analysis (CCA) was utilized for the per-protocol estimate (i.e., analysis of participants according to the completion of the assigned treatment).

**Table 1:** Baseline characteristics of erythrodermic psoriasis patients identified in the study (*N* = 311).

Characteristics	First analysis			Second analysis		
	Secukinumab cohort (*N* = 160)	CST cohort (*N* = 151)	*p* value	Secukinumab cohort (*N* = 101)	CST cohort (*N* = 101)	*P* value
Age (years), mean ± SD	47.2 ± 15.7	49.9 ± 17.6	0.143	48.2 ± 16.5	48.5 ± 17.5	0.908
Sex, *n* (%)			0.061			0.508
Male	128 (80.0)	107 (70.9)		79 (78.2)	75 (74.3)	
Female	32 (20.0)	44 (29.1)		22 (21.8)	26 (25.7)	
Marital status			**0.010**			1.000
Not Married	37 (23.1)	18 (11.9)		15 (14.9)	15 (14.9)	
Married	123 (76.9)	133 (88.1)		86 (85.1)	86 (85.1)	
Education level			**<0.001**			0.098
≤ Secondary School	46 (28.7)	91 (60.3)		33 (32.7)	41 (40.6)	
High school	75 (46.9)	37 (24.5)		52 (51.5)	37 (36.6)	
College & above	39 (24.4)	23 (15.2)		16 (15.8)	23 (22.8)	
Smoking, *n* (%)			0.458			0.140
Never	101 (63.1)	103 (68.2)		71 (70.3)	69 (68.3)	
Quit	15 (9.4)	9 (6.0)		10 (9.9)	4 (4.0)	
Current smoking	44 (27.5)	39 (25.8)		20 (19.8)	28 (27.7)	
Family history of psoriasis, *n* (%)			**0.004**			1.000
Yes	29 (18.1)	11 (7.3)		11 (10.9)	11 (10.9)	
No/unknown	131 (81.9)	140 (92.7)		90 (89.1)	90 (89.1)	
Having psoriatic arthritis, *n* (%)	52 (32.5)	31 (20.5)	**0.017**	26 (25.7)	27 (26.7)	0.873
History of Cancer, *n* (%)	4 (2.5)	5 (3.3)	0.670	0 (0.0)	4 (4.0)	0.130
History of systemic treatment, *n* (%)	91 (56.9)	94 (62.3)	0.334	59 (58.4)	68 (67.3)	0.190
Having underlying diseases, *n* (%)			0.921			0.614
Yes	38 (23.8)	35 (23.2)		25 (24.8)	20 (19.8)	
No	103 (64.4)	100 (66.2)		66 (65.3)	68 (67.3)	
Unknown	19 (11.9)	16 (10.6)		10 (9.9)	13 (12.9)	
Duration, medium (IQR)	9.5 (0.0, 18.8)	9.0 (1.0, 20.0)	0.682	10.0 (0, 19.0)	8.0 (2.0, 20.0)	0.840
BMI (kg/m^2^), medium (IQR)	24.0 (22.0, 26.0)	23.9 (22.0, 25.7)	0.984	24.2 (22.2, 26.0)	23.9 (22.7, 26.0)	0.722
DQLI score, medium (IQR)	14.0 (9.0, 21.0)	13.0 (9.0, 18.0)	0.092	14.5 (8.0, 21.0)	13.5 (9.0, 18.0)	0.309
BSA score, medium (IQR)	74.0 (30.0, 90.0)	70.0 (18.0, 90.0)	0.380	72.0 (30.0, 81.7)	62.5 (16.5, 90.0)	0.579
PASI score, medium (IQR)	26.5 (14.4, 41.0)	23.3 (13.2, 44.0)	0.840	24.7 (15.2, 38.7)	22.8 (11.1, 48.9)	0.935

Bold: significant values. *N*, number; SD, standard deviation; IQR, interquartile range; CST, conventional systemic therapy; PASI, Psoriasis Area and Severity Index; BSA, Body Surface Area; DLQI, Dermatology Life Quality Index; BMI, Body Mass Index.

### Efficacy and quality of life

Before and after PSM, secukinumab recipients demonstrated a faster response compared to CSTs, with significantly higher PASI50 (before: *p* = 0.022, after: *p* = 0.046), PASI90 (before: *p* = 0.036, after: *p* = 0.025), and BSA50 (before: *p* = 0.012, after: *p* = 0.015) rates achieved at week 4. However, before matching, the secukinumab cohort had significantly higher DLQI0/1 rates than the CST cohort at W4 (*p* = 0.035) and W12 (*p* = 0.04). Additionally, similar changing patterns trends were also observed for most efficacy indicators over time up to W24 in all recipients, but with no statistical differences. The magnitude of improvement over time was mostly greater in patients treated with secukinumab compared to CST; however, after PSM, statistically significant differences were observed only at W12 for PASI scores (*p* = 0.012), at W12 for BSA scores (*p* = 0.008), and at W4 for DLQI scores (*p* = 0.046). The comparative results of key efficacy and QoL outcomes at each time point, before and after matching, are detailed in Table 2 Comparisons of PASI, BSA, and DLQI scores at each time point after PSM are outlined in Table 3.

**Table 2:** Key efficacy and quality-of-life outcomes at follow-up time points in erythrodermic psoriasis patients treated with secukinumab and conventional systemic therapies.

	Before matching			After matching		
	Secukinumab cohort	CST cohort	*P* value	Secukinumab cohort	CST cohort	*P* value
PASI50 response, *n*/*N* (%)						
Week 4	118/160 (73.8)	93/151 (61.6)	**0.022**	77/101 (76.2)	64/101 (63.4)	**0.046**
Week 12	49/59 (83.1)	49/59 (83.1)	1.000	29/34 (85.3)	28/34 (82.4)	0.742
Week 24	24/29 (82.8)	28/32 (87.5)	0.602	12/15 (80)	13/15 (86.7)	0.500
PASI75 response, *n*/*N* (%)						
Week 4	92/160 (57.5)	74/151 (49.0)	0.166	62/101 (61.4)	51/101 (50.5)	0.119
Week 12	44/59 (74.6)	44/59 (74.6)	1.000	27/34 (79.4)	24/34 (70.6)	0.401
Week 24	23/29 (79.3)	25/32 (78.1)	0.910	12/15 (80)	12/15 (80.0)	0.674
PASI90 response, *n*/*N* (%)						
Week 4	59/160 (36.9)	39/151 (25.8)	**0.036**	41/101 (40.6)	26/101 (25.7)	**0.025**
Week 12	36/59 (61.0)	35/59 (59.3)	0.851	22/34 (64.7)	20/34 (58.8)	0.618
Week 24	17/29 (58.6)	18/32 (56.3)	0.852	7/15 (46.7)	7/15 (46.7)	0.642
BSA50 response, *n*/*N* (%)						
Week 4	103/160 (64.4)	76/151 (50.3)	**0.012**	69/101 (68.3)	52/101 (51.5)	**0.015**
Week 12	46/59 (78.0)	45/59 (76.3)	0.827	27/34 (79.4)	27/34 (79.4)	1.000
Week 24	23/29 (79.3)	28/32 (87.5)	0.388	12/15 (80)	13/15 (86.7)	0.500
BSA75 response, *n*/*N* (%)						
Week 4	79/160 (49.4)	58/151 (38.4)	0.052	53/101 (52.5)	43/101 (42.6)	0.159
Week 12	40/59 (67.8)	39/59 (66.1)	0.845	24/34 (70.6)	23/34 (67.6)	0.793
Week 24	21/29 (72.4)	23/32 (71.9)	0.963	12/15 (80)	12/15 (80.0)	0.650
DQLI0/1 response, *n*/*N* (%)						
Week 4	66/160 (41.3)	45/151 (29.8)	**0.035**	46/101 (45.5)	37/101 (36.6)	0.198
Week 12	37/59 (63.8)	26/59 (44.8)	**0.040**	18/34 (54.5)	12/34 (36.4)	0.138
Week 24	21/29 (72.4)	16/32 (51.6)	0.098	11/15 (73.3)	6/15 (40.0)	0.070

Bold: significant values. *N*, number; IQR, interquartile range; CST, conventional systemic therapy; PASI, Psoriasis Area and Severity Index; BSA, Body Surface Area; DLQI, Dermatology Life Quality Index; BMI, Body Mass Index.

**Table 3:** Comparison of Psoriasis Area and Severity Index (PASI), Body Surface Area (BSA), and Dermatology Life Quality Index (DLQI) score between Secukinumab recipients and conventional systemic therapies (CSTs) recipients, after propensity-score matching (PASI: score range 0–72, BSA: percentage, DLQI: score range 0–30).

	Secukinumab cohort, medium (IQR)	CST cohort medium (IQR)	*P* value
PASI score			
Week 0 (*n* = 101 matched pairs)	14.5 (8.0, 21.0)	13.5 (9.0, 18.0)	0.309
Week 4 (*n* = 101 matched pairs)	2.7 (0.1, 10.5)	3.4 (1.3, 12.5)	0.144
Week 12 (*n* = 34 matched pairs)	1.1 (0, 2.3)	3.0 (0.8, 6.9)	**0.012**
Week 24 (*n* = 15 matched pairs)	3.1 (0.6, 6.3)	3.0 (1.2, 6.2)	0.713
BSA score			
Week 0 (*n* = 101 matched pairs)	72.0 (30.0, 81.7)	62.5 (16.5, 90.0)	0.579
Week 4 (*n* = 101 matched pairs)	5.0 (1.0, 30.0)	9.0 (0, 39.0)	0.457
Week 12 (*n* = 34 matched pairs)	1.0 (0, 5.0)	7.0 (1.0, 20.5)	**0.008**
Week 24 (*n* = 15 matched pairs)	5.0 (0.5, 22.0)	5.0 (0.5, 30.0)	0.967
DQLI score			
Week 0 (*n* = 101 matched pairs)	24.7 (15.2, 38.7)	22.8 (11.1, 48.9)	0.935
Week 4 (*n* = 101 matched pairs)	2.0 (0, 8.0)	4.0 (1.0, 9.0)	**0.046**
Week 12 (*n* = 34 matched pairs)	1.0 (0, 5.5)	4.0 (0, 9.0)	0.088
Week 24 (*n* = 15 matched pairs)	1.0 (0, 3.0)	3.0 (0, 10.0)	0.148

Bold: significant values.

### Safety

Throughout the 24-week observation period, eight AEs were documented in eight distinct patients (2.57%) of both cohorts. Among them, five were from the secukinumab group (3.12%) and three from the CST group (1.98%). The majority of reported AEs were mild and self-limiting in two groups. No AEs were recorded that required prolonged hospitalization or presented potential life-threatening consequences, thereby avoiding their classification as serious AEs (SAEs). However, two AEs who underwent secukinumab were categorized under the MedDRA high-level term of *fungal infection disorders*. The result details are listed in Table 4.

**Table 4:** Treatment-related adverse events (AEs) observed during patient’s follow-up.

Category and description	Treatment	Delay after starting Treatment	treatment maintenance	Outcome	SAE/AE
Fungal infection disorders (jock itch)	Secukinumab	Week 10	Yes	Recovery	AE
Fungal infection disorders (tinea corporis)	Secukinumab	Week 3	Yes	Recovery	AE
Skin and subcutaneous tissue disorders					
Urticaria	Secukinumab	Week 5	Yes	Recovery	AE
Allergic dermatitis	Secukinumab	Week 13	Yes	Recovery	AE
Others					
Abnormal kidney function	Secukinumab	Week 3	Yes	Not recovery	AE
Muscle soreness	Retinoids	Week 1	Yes	Recovery	AE
Dry mucous membranes	Retinoids	Week 2	Yes	Unknow	AE
Liver damage (elevated transaminases)	Retinoids	Week 11	Yes	Not Recovery	AE

AE, adverse events; SAE, serious AE.

## Discussion

Differences in proinflammatory cytokine expression are observed in various clinical subtypes of psoriasis. Literature suggests that the immunopathogenesis of EP involves inflammation with Th1/Th17/TNF-skewing and mild Th2 (IL-4, IL-10, IL-13, and IL-25) upregulation, as well as notable increases in epidermal-development markers (18, 19). Disease severity in EP was strongly correlated with Th17 markers (18). Similar tendencies were observed in immunohistochemical analysis, which revealed that the primary T-cell subset present in EP lesions is Th17. Increased IL-17 mRNA expression and elevated serum IL-17 levels have been demonstrated in patients with EP (12). This leads to the production of antimicrobial peptides, cytokines, and chemokines, which further enhance inflammation (11–13). These results indicate that IL-17A is a crucial pathway in both plaque psoriasis and EP, potentially being the most essential cytokine in EP. As a fully human monoclonal IgG1κ antibody that neutralizes IL-17A, secukinumab has been shown to decrease IL-17 production and modulate gene expression associated with various cytokines and chemokines in chronic plaque psoriasis, including IL-17A, IL-21, IL-22, CCL20, KRT16, and DEFB4 (20). However, in EP patients, the cytokine pathways are not entirely consistent with those in chronic plaque psoriasis, and the molecular regulatory mechanisms of secukinumab require further investigation.

Secukinumab has exploratory applications in EP, providing some clinical experience and anecdotal evidence. The largest case series recently, a multi-center, international, retrospective pilot study that enrolled 13 EP patients receiving secukinumab, reported that PASI90/100 responses were achieved by 5 (38.5%) and 4 (30.8%) patients at week 16 (4). Similarly, a real-life study that enrolled 10 patients reported that PASI75/90 responses were achieved by 5 (50.0%) and 3 (30.0%) patients at week 8 (21). In a head-to-head cohort study involving 15 EP patients, ixekizumab achieved faster PASI90 at 12 weeks compared to secukinumab. However, secukinumab demonstrated statistically superior results at the endpoint, with 82% of patients achieving PASI-90 and 54% achieving PASI-100 at week 48 (22). Additionally, several significant results regarding secukinumab for pediatric EP were obtained from case studies (23, 24). For the low incidence rate of EP and the necessity for immediate intervention, large-sample RCTs remain unavailable, posing challenges in establishing standardized treatment protocols supported by high-level clinical evidence.

It has been reported that the prevalence of EP is higher in Asians and Hispanics compared to Caucasians, this may be due to socioeconomic differences and healthcare accessibility (2). Due to the diversity in incidence rate, NCRC-DID documented a relatively substantial number of Chinese patients with this rare psoriasis subtype. To our knowledge, this study is the first comparative cohort study to investigate the real-world effectiveness of secukinumab compared to traditional systemic therapies for EP, including 311 patients and utilizing the PSM approach. Consistent with prior studies, our findings provide additional evidence supporting the relative superiority of secukinumab compared to other systemic agents in EP. There was a statistically significant improvement in clinical symptoms and signs from baseline in both EP cohorts. The secukinumab cohort demonstrated a faster response compared to CST, with significantly higher PASI50, PASI90, and BSA50 rates achieved at week 4 in both the unadjusted and PSM analysis. Assessment of DLQI0/1 revealed that secukinumab also tended to result in quicker and greater improvement in the quality of life for EP. Moreover, the reduction in PASI, BSA, and DLQI scores of the secukinumab cohort was numerically more than that of the CST cohort at all follow-up timepoints, implying a potentially higher degree of clinical response. The AEs observed in our study were consistent with the established safety profile of the biologics used (10, 14, 25). These AEs did not recur in subsequent treatment cycles.

The retention rate is often used to measure adherence and evaluate the long-term effectiveness, safety, and real-world utility of medications. In this study, the drug survival of secukinumab in patients with erythrodermic psoriasis (EP) at Week 8 and Week 24 was 36.9% (59/160) and 18.1% (29/160), respectively. These rates are lower compared to plaque psoriasis (PP) in similar studies, which reported 12-month survival rates of 83% and 4-year survival rates of 74.7% (26, 27). This discrepancy may be attributed to several factors. The more localized and IL-17-centric nature of PP allows for better outcomes with secukinumab. In contrast, the systemic and multifactorial nature of EP, involving additional inflammatory pathways such as TNF-skewing and Th2 upregulation (18, 19), suggests that EP patients often require combination therapies or alternative biologics to achieve and maintain disease control, which may contribute to higher rates of treatment discontinuation. Furthermore, EP patients are more likely to have a history of biologics failures and comorbidities, further complicating treatment outcomes.

The primary limitation pertains to the extent of missing outcome data. Although missing outcome data was evenly distributed across groups, the available information did not provide sufficient clarity to discern whether recipients were lost to follow-up due to clinical improvement, nonresponse, side effects, financial burden, or other factors. Adjusted comparative studies could offer prompt insights and complement the comparative effectiveness gap in treatments for EP. However, this approach has inherent contingency and selection bias and is not a substitute for direct comparison RCTs.

## Conclusion

This comparative study using PSM was adjusted for potential confounding factors to reduce bias due to reverse causation and provided preliminary evidence regarding the comparative effectiveness of secukinumab versus CST in EP treatments. Compared to CST, secukinumab recipients tend to experience a more rapid response and achieve greater improvement, which is demonstrated by higher rates of achieving PASI50, PASI75, and DLQI 0/1 at week 4, as well as greater improvements in PASI, BSA, and DLQI scores throughout the follow-up period.

## Data Availability

The original contributions presented in the study are included in the article/supplementary material, further inquiries can be directed to the corresponding author.

## Footnotes

^1^ https://derm.pku.edu.cn
